# The role of C5a-C5aR1 axis in bone pathophysiology: A mini-review

**DOI:** 10.3389/fcell.2022.957800

**Published:** 2022-08-08

**Authors:** Anna Ruocco, Anna Sirico, Rubina Novelli, Silvia Iannelli, Shane Vontelin Van Breda, Diego Kyburz, Paul Hasler, Andrea Aramini, Pier Giorgio Amendola

**Affiliations:** ^1^ R&D, Dompé Farmaceutici SpA, Naples, Italy; ^2^ R&D, Dompé Farmaceutici SpA, Milan, Italy; ^3^ Departement Biomedizin, University of Basel, Basel, Switzerland; ^4^ Division of Rheumatology, Kantonsspital Aarau AG, Aarau, Switzerland; ^5^ R&D, Dompé Farmaceutici SpA, L’Aquila, Italy

**Keywords:** C5a, C5aR1, bone, rheumatoid arthritis, osteoclasts, osteoblasts

## Abstract

Bone remodeling is a physiological, dynamic process that mainly depends on the functions of 2 cell types: osteoblasts and osteoclasts. Emerging evidence suggests that complement system is crucially involved in the regulation of functions of these cells, especially during inflammatory states. In this context, complement component 5a (C5a), a powerful pro-inflammatory anaphylatoxin that binds the receptor C5aR1, is known to regulate osteoclast formation and osteoblast inflammatory responses, and has thus been proposed as potential therapeutic target for the treatment of inflammatory bone diseases. In this review, we will analyze the role of C5a-C5aR1 axis in bone physiology and pathophysiology, describing its involvement in the pathogenesis of some of the most frequent inflammatory bone diseases such as rheumatoid arthritis, and also in osteoporosis and bone cancer and metastasis. Moreover, we will examine C5aR1-based pharmacological approaches that are available and have been tested so far for the treatment of these conditions. Given the growing interest of the scientific community on osteoimmunology, and the scarcity of data regarding the role of C5a-C5aR1 axis in bone pathophysiology, we will highlight the importance of this axis in mediating the interactions between skeletal and immune systems and its potential use as a therapeutic target.

## Introduction

Complement component 5a (C5a) is one of the most potent inflammatory proteins of the complement system. It results from the cleavage of the precursor protein C5 by the enzyme C5 convertases and binds to C5a receptor 1 (C5aR1 or CD88) (Ehrnthaller et al., 2011) and C5aR2 (C5a receptor-like two or C5L2), which are expressed on the surface of immune cells and, also, ubiquitously on other cell types (Monk et al., 2007). As complement component 3a (C3a), C5a is an anaphylatoxin, whose activation leads to clearance of foreign cells, vasodilation, chemotaxis of inflammatory cells, cytokine and chemokine release, oxidative burst of immune cells (Guo and Ward, 2005) and induction and amplification of inflammatory reactions (Ricklin et al., 2010).

Besides its crucial role in the immune system-mediated protection from internal and external threats, C5a and its widely expressed receptors are also emerging as important players in different pathophysiological processes (Zheng et al., 2019; Carvelli et al., 2020; Giorgio et al., 2021; Wu et al., 2022). In addition to its activation in response to pathogens in fact, C5a formation can be also triggered by complement-independent enzymes, such as thrombin, neutrophil elastase and a macrophage serine protease, which have C5 convertase (C5a-generating) activity (Huber-Lang et al., 2015) and can thus activate C5a in tissues in response to several stimuli. Among the processes and tissues that are targets of C5a functions, growing evidence has shown that C5a-C5aR axis has an impact on the skeletal system, where it regulates bone metabolism and turnover both under physiological and pathophysiological conditions (Modinger et al., 2018).

In this mini-review, we will discuss the role of the C5a-C5aR1 axis in bone physiology and pathology, focusing on its involvement in the pathogenesis of inflammatory disorders of the skeletal system, as in particular rheumatoid arthritis, and also osteoporosis and cancer metastasis to the bones.

## The role of C5a in bone physiology

Bone is an extremely dynamic tissue that undergoes continuous remodeling during the lifetime, and this process is carried out by three types of cells (Ponzetti and Rucci, 2019): osteoclasts, which are bone-resorbing cells deriving from stem cells of the macrophage-hematopoietic lineage; osteoblasts, that are bone-forming cells (Matsuoka et al., 2014); and osteocytes, which are former osteoblasts buried in the bone mineral matrix (Metzger and Narayanan, 2019).

**FIGURE 1:**
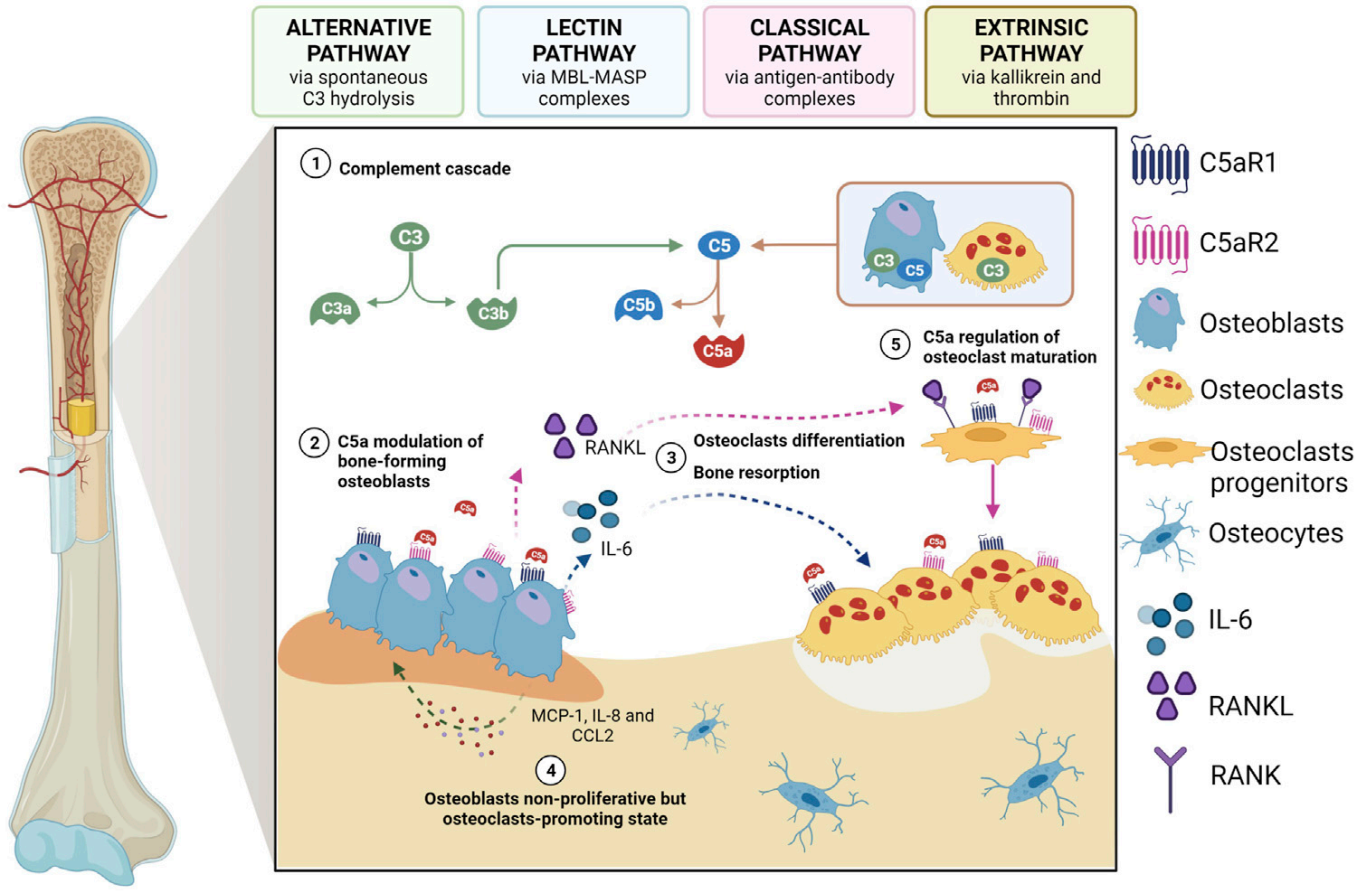
The role of C5a as a modulator of osteoblast-osteoclast interplay. 1) Activated complement system leads to the generation of C5a which can bind to C5aR1/2 on osteoblasts; 2) once activated by C5a, osteblasts start to release IL-6 and RANKL, thus inducing 3) osteoclastogenesis and bone resorption as well as differentiation of osteoclast progenitors; 4) activated osteoblasts can also secrete other chemokines and cytokines, such as MCP-1, CCL2 and IL-8, which in turn act on osteoblasts inducing a non-proliferative osteoclast-promoting state; 5) C5a also regulates the first phase of osteoclastogenesis maturation. Created with BioRender.com.

In physiological conditions, studies have been indicating a direct involvement of complement system in bone development and homeostasis. In support of this, osteoblasts express both C3 and C5, while osteoclasts express only C3, but both cells are able to cleave C5 (and not C3) and generate C5a. Moreover, the receptors C3aR, C5aR1 and C5aR2 are expressed on both cell types (Ignatius et al., 2011a) (Figure 1). Multiple complement components, including C3 and C5, were described to have a characteristic expression pattern in distinct zones of the epiphyseal growth plate, suggesting a role for complement during bone development (Andrades et al., 1996). The specific expression of C5 in the hypertrophic zone of the growth plate together with the evidence that C5-deficient mice have thicker epiphyseal growth plates, potentially due to delayed endochondral ossification, suggest in fact that C5 requirement is important during bone formation and longitudinal bone growth (Ehrnthaller et al., 2013). Finally, C3a, C3aR, and C5aR have shown to be crucial for the regulation of calcified cartilage matrix degradation mediated by osteoclasts, the formation — but not the resorption activity — of which is significantly enhanced in the presence of C3a and C5a (Ignatius et al., 2011a; Kovtun et al., 2017).

Although no observations on bone malformations in development or bone density have been published in humans carrying C5 deficiency, and this is most probably due to the rarity of such condition and its lethality (i.e., Leiner`s disease, which is particularly fatal if not corrected at infancy) (Guenther, 1983), the relevance of the C5a-C5aR axis in bone formation and regulation of its structure has been confirmed in preclinical studies. Twelve-week-old C5aR1-knockout (ko) and C5aR2-ko mice showed in fact a higher bone-mass phenotype compared to wild-type controls, and this effect was more pronounced in C5aR1-ko mice, where it was associated with decreased osteoclasts in trabecular bone (Kovtun et al., 2017). Moreover, pharmacological inhibition of C5a pathway during embryo-foetal development using avacopan, a small molecule C5aR antagonist (Harigai and Takada, 2022), induced an increased incidence of skeletal variations in hamsters, further confirming the role of the complement during bone development (European Medicines Agency, 2022).

In the adult skeleton, osteoclasts and osteoblasts, and their respective mesenchymal and haematopoietic precursors, closely interact and communicate in a fine-tuned balance that is a prerequisite for bone homeostasis. The C5a-C5aR1 axis plays a role in this context, as it can regulate the expression of different mediators that are involved in this process (Modinger et al., 2018). C5a can, for example, modulate the release of interleukin (IL)-6 from osteoblasts (Pobanz et al., 2000), thus inducing osteoclastogenesis and bone resorption (Ishimi et al., 1990), and this can happen *via* the induction of the expression of receptor activator of nuclear factor kappa-B (RANK) ligand (RANKL) in osteoblasts (Ishimi et al., 1990) or without its induction (Figure 1). Secreted by osteoblasts, RANKL stimulates osteoclastogenic differentiation by binding to its receptor RANK on the membrane of osteoclast-committed monocytes (Lacey et al., 1998), while other chemokines (e.g., monocyte chemoattractant protein-1 (MCP-1; CCL2) and cytokines, including IL-8 (CXCL8), act on osteoblasts inducing a non-proliferative but osteoclast-promoting state (Pathak et al., 2015) (Figure 1). Indeed, *in vitro* IL-8 stimulation has been shown to enhance IL-6 gene expression and protein production by human osteoblasts obtained from bone biopsies, indicating that IL-8-stimulated osteoblasts can produce factors that are essential for osteoclast formation (Pathak et al., 2015). Notably, the role of C5aR in regulating the first phases of osteoclast maturation has also been recently demonstrated in RAW264.7 cells (D'Angelo et al., 2020), which are murine monocytes/macrophages that upon treatment with RANKL can form multinucleated and functionally active osteoclast-like cells. Indeed, in these cells, both C5aR downregulation and antagonism—by C5aR antagonist PMX-53 and two newly synthesized allosteric C5aR antagonists, DF2593A and DF3016A—inhibited osteoclast maturation, as demonstrated by the reduced RANKL-triggered transcription of the most important osteoclast differentiation markers, such as NFATc1, MMP-9, cathepsin-K, and TRAP. Interestingly, it was observed that, as osteoclast differentiation progressed, C5aR mRNA expression decreased, with a consequent less impact of C5aR on the regulation of later events of osteoclast fusion (D'Angelo et al., 2020).

C5a can also induce the production of macrophage-colony stimulating factor (M-CSF) and plays a chemotactic role, together with the anaphylatoxin C3a, for immune cells, human mesenchymal stem cells (MSCs) (Schraufstatter et al., 2009; Moll et al., 2011), osteoclast and osteoblast precursors, and, at an even higher rate, for mature osteoblasts (Ignatius et al., 2011b).

Thus, complement proteins, and especially C5a-C5aR1 axis, are directly and indirectly involved in the physiology of the bone tissue during development and homeostasis (Pobanz et al., 2000; Ignatius et al., 2011a), as well as in its pathology, especially when a pro-inflammatory status develops in the bone environment (DiScipio et al., 2013). Indeed, the state of complement activation has been found to play a role in the development and progression of several bone-related inflammatory disorders, and in particular rheumatoid arthritis (RA), which is an excellent model of osteoimmunology because of the extensive involvement of the immune system in its pathogenesis, and also osteoporosis and cancer bone metastasis.

## C5a in Rheumatoid Arthritis

Rheumatoid Arthritis (RA) is a systemic autoimmune disease that affects 0.24% of the general population worldwide, according to the Global Burden of Disease 2017 study (Disease, 2018; Safiri et al., 2019), with a higher prevalence in females than males. The risk to develop RA is age-dependent, with an incidence peak between 65 and 80 years of age and a lifetime risk of 1.7% in men compared to 3.6% in women (Crowson et al., 2011; Eriksson et al., 2013; World Health Organization, 2018). RA main clinical manifestations include pain and swelling of hands, wrists, and foot and knee (polyarthritis) joints. Some patients may also develop manifestations in other organs, even with no articular involvement, such as interstitial lung disease (ILD), pericarditis, pleural effusion, or bronchiectasis (Littlejohn and Monrad, 2018; Conforti et al., 2021). Treatment of RA is aimed at reducing joint inflammation and pain, preventing joint destruction and maximizing joint function: first-line RA treatments are nonsteroidal anti-inflammatory drugs (NSAIDs) (Bullock et al., 2018), and corticosteroids are also used, but for a short period of time and at low doses due to their greater side effects (Lim and Bolster, 2019). In addition, there are the disease-modifying antirheumatic drugs (DMARDs) that can be synthetic (small chemical molecules given orally) or biologic (proteins administered parenterally).

The etiology of RA remains unknown; however, it is generally accepted that it results from the combined effects of patients’ genotype and environment (Klareskog et al., 2006). In RA patients, the persistent articular inflammation is driven by the proliferation of synovial tissue fibroblasts and by the infiltration of immune cells, such as T and B lymphocytes, neutrophils and monocytes, and induces the formation of the pannus, an abnormal synovial tissue, which invades and destroys local articular structures. The infiltrating cells of the pannus express pro-inflammatory cytokines, chemokines (as IL-6, IL-8, TNF-alfa and IL-1) and matrix metalloproteinases, such as MMP-2 and MMP-9, that contribute to a progressive distruction of both cartilage and bone (McInnes et al., 2016; Zhang, 2021).

**FIGURE 2:**
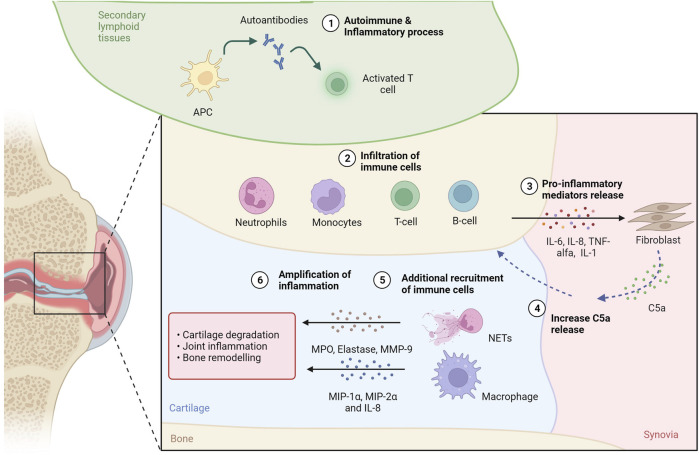
C5a in rheumatoid arthritis. In secondary tissues of RA patients, production of autoantibodies 1) activates inflammation and attracts immune cells 2), such as T and B lymphocytes, neutrophils and monocytes, to the inflammation site. The release of proinflammatory molecules 3) drives the proliferation of synovial tissue fibroblasts, which can contribute to the increase of C5a levels in the synovial fluid (SF) 4). C5a contributes to additional immune cell recruitment 5) and activation: neutrofils can undergo NETosis releasing proteaseses (i.e., MPO, elastase, MMP-9), while macrophages can release inflammatory proteins (i.e., MIP-1α, MIP-2α and IL-8). The release of proteases and proinflammatory proteins supports cartilage degradation, joint inflammation and bone remodeling, amplyfing the inflammatory state 6). Created with BioRender.com.

Synovitis, swelling and joint damage are caused by a complex autoimmune and inflammatory process mediated by both the innate and adaptive immune systems (Gibofsky, 2014). Inflammatory cell recruitment into the synovial fluid and tissue occurs as a result of the organized action of chemoattractants (e.g., RANTES) and macrophage inflammatory proteins (i.e., MIP-1α, MIP-2α and IL-8) produced by activated macrophages, synovial fibroblasts, and other cells in the inflamed joint. The increase in inflammatory cells is also due to the chemotactic action of complement activation products, such as C5a, the level of which is increased in synovial fluid (SF) compared to plasma concentration (Moxley and Ruddy, 1985; Jose et al., 1990; Boackle, 2003) (Figure 2).

C5a is a potent neutrophil chemoattractant and priming agent that induces oxidative bursts and release of effector molecules from neutrophils and of cytokines from monocytes and macrophages (Hogasen et al., 1995). In RA, neutrophils and macrophages are the cells that primarily express C5aR (Hornum et al., 2017), also described as a key initiator of neutrophil adhesion (Miyabe et al., 2017). Interestingly, when neutrophils *in vitro* were exposed to GM-CSF and C5a, which are both abundant factors in RA, neutrophil extracellular traps (NETs) formation has been observed. NETs are networks of modified histones (citH3), DNA fiber and antimicrobial proteins (MPO, elastase, others) released by neutrophils to entrap and facilitate the killing of pathogens in a process named NETosis (Disease, 2018). In RA pathogenesis, the role of NETosis has been investigated (Crowson et al., 2011; Eriksson et al., 2013), demonstrating that when NETosis occurs, citrullinated proteins are released and, when recognized by anti-citrullinated protein antibodies (ACPAs), initiate and propagate the aberrant immune responses and inflammation that is characteristic of RA (World Health Organization, 2018; Safiri et al., 2019). NETs have also been shown to provide a scaffold for the alternative complement pathway, leading to C5a generation. In addition, properdin, which is an essential positive regulator of the complement pathway that allows for the formation of the C3 convertase C3bBb of the alternative pathway and thus the formation of C5a, has also been observed to be present on NETs (Wang et al., 2015).

C5a/C5aR1 axis acts also on the luminal endothelium surface of the joint vasculature, where immune complexes that deposit in the joint can trigger C5a generation. Interestingly, the inhibition of NETosis by DNase one abrogated C5a production, ultimately reducing endothelial cell damage *in vitro* (Schreiber et al., 2017). C5a then binds to heparan sulfate proteoglycan (HSPG) on synovial endothelium, leading to the arrest of neutrophils *via* ß_2_ integrin activation. This signalling causes the release of leukotriene B_4_ (LTB_4_), initiating autocrine/paracrine actions *via* the BLT1 receptor and allowing neutrophils to move from the blood vessel lumen into the interstitium. Neutrophils in the joint space can then propagate their survival *via* CXCL2-CXCR2 signaling (Sadik et al., 2018).

Thus, not only is C5a responsible for NET formation, but it can also be important for diapedisis into the joint, where the NETs can further damage cartilage and bone. Apart from causing damage in RA joints, NETs can further provide a scaffold for the alternative pathway, increasing formation of C5a and acting as an amplification loop for C5a production, recruitment of neutrophils into the joint, NET production, and cartilage and bone damage (Figure 2). Targeting C5a or the C5aR might thus be a viable solution for modulating NET formation in RA, thus preventing the destruction of cartilage (Carmona-Rivera et al., 2020) and bone (O'Neil et al., 2020) and reducing endothelial cell damage (Schreiber et al., 2017). In agreement, both genetic ablation or pharmacological inhibition of the C5a-C5aR axis improved arthritis or prevented the disease in animal studies (Wang et al., 1995; Goodfellow et al., 2000; Ames et al., 2001; Grant et al., 2002; Ji et al., 2002; Woodruff et al., 2002; Katschke et al., 2007; Banda et al., 2012). This strategy could be effective as it can target also the pro-osteoclastogenic effect of C5a that in the inflammed bone, as happens in RA and bone healing, enhances the inflammatory response of osteoblasts and increases osteoclast formation (Hornum et al., 2017; Modinger et al., 2018).

## Other diseases

### C5a/C5aR1 axis in osteoporosis-related bone fracture

Osteoporosis is a bone disease characterized by a decrease of bone mineral density and bone mass (Nikolaou et al., 2009). The etiologic determinants of osteoporosis include endocrine and metabolic conditions and mechanical factors, including sex, body size, race, family history, changes of hormones (postmenopausal hormonal condition, pregnancy), diet (insufficient vitamin D and calcium intake), lifestyle and long term use of certain medications (Yun and Lee, 2004). Specific pathologies, such as gastrointestinal diseases, RA, certain types of cancer, HIV/AIDS and anorexia nervosa, have also been considered as triggers of osteoporosis (Ginaldi et al., 2005). In osteoporosis patients, risk of bone fractures is raised and frequently associated with healing complications, prolonged hospitalization, and increased morbidity and mortality (Nikolaou et al., 2009; von Rüden and Augat, 2016; Giannoudis et al., 2007).

Emerging clinical and molecular data, along with a growing understanding of bone remodeling processes, have suggested that inflammation is crucially involved in bone turnover (Lorenzo, 2000) and healing, and thus in the onset of osteoporosis and recovery after fractures in these patients (Kiecolt-Glaser et al., 2003). Following an isolated fracture, the complement system critically modulates bone regeneration and healing (Huber-Lang et al., 2015), particularly through the C5a/C5aR1 axis (Bergdolt et al., 2017). C5a in fact is a strong activator of mast cells and triggers the rapid release of pre-formed granular factors (Moon et al., 2014; Erdei et al., 2004; el-Lati et al., 1994) that mediate osteoclastogenic effects (Kroner et al., 2017). C5aRs, on the other hand, are strongly expressed in the fracture callus, not only by immune cells, but also by bone cells and chondroblasts (Huber-Lang et al., 2015), and the relative spatial expression and functionality of the two C5a receptors on bone and immune cells during the healing period crucially influences post-fracture outcome (Ehrnthaller et al., 2013).

Genetically modified animal models have allowed to investigate and demonstrate the crucial role of the C5a/C5aR axis in fracture healing (Ehrnthaller et al., 2013). C5-deficient mice for example displayed a reduced volume and mechanical properties in fracture calluses, indicating impaired healing (Ehrnthaller et al., 2013). In addition, C5aR1 knockout mice showed a decrease of early inflammation in the fracture callus but also a disturbed final healing outcome in late healing stages, including the cartilage-to-bone transition (Kovtun et al., 2017). Interestingly, similarly disturbed fracture healing was also observed in C5aR2-ko mice subjected to a femur fracture (Kovtun et al., 2017). On the other hand, osteoblast-specific C5aR1-overexpression disturbed fracture healing in mice subjected to a femur fracture, with or without the induction of an additional systemic inflammation by thoracic trauma, diminishing mechanical properties of the healed femur, reducing bone content of the fracture callus, and increasing impairment following severe trauma compared to wild-type littermates (Bergdolt et al., 2017). These data strongly suggest that the C5a/C5aR axis directly affect osteoblasts activity on bone healing and regeneration, exerting a fine and tight regulation of fracture healing during the entire process (Bergdolt et al., 2017).

### C5a, bone cancer and neutrophil extracellular traps

Bone tumors represent a real challenge in oncology (Ferguson and Turner, 2018). They can grow as primary cancers or as consequence of metastatic colonization (Ferguson and Turner, 2018). Primary bone cancers are rare, accounting for about 0.2% of all malignancies worldwide, while secondary bone tumors represent one of the most common type of metastasis following advanced stages of lungs, liver, breast and prostate cancers (Coleman, 2001; Pullan and Budh, 2021). There are two main types of bone metastasis: the osteolytic lesions that are caused by the cancer cell-induced activation of osteoclastogenesis, which results in the complete destruction of bone and its substitution with cancer cells (Guise et al., 2006); and the osteosclerotic lesions, which are instead caused by aberrant osteoblast activation that produces low quality extra bone tissues (Ibrahim et al., 2010). Notably, the latter are also characterized by an increased osteoclast activity and bone resorption, which is needed to create the space for cancer cells to growth (Maurizi and Rucci, 2018).

C5a plays a crucial role in regulating tumor growth, metastasis, and drug resistance (Ajona et al., 2019). Expression of C5aR1 on cancer cells enhances their motility, invasiveness and epithelial to mesenchymal transition (Nitta et al., 2013; Maeda et al., 2015; Hu et al., 2016). In non-small-cell lung cancer (NSCLC) for example, higher C5aR1 levels in the primary tumor predict bone metastasis and result in decreased overall survival and relapse free survival (Ajona et al., 2018a). Accordingly, activation of the C5a/C5aR1 axis induced a pro-metastatic phenotype in lung cancer cells in culture, while favoring bone colonization *via* regulation of CXCL16 release, which in turns promotes a proosteoclastogenic environment in bone metastasis (Ajona et al., 2018a). In addition to cancer cells, osteoblasts also highly express C5aR1 (Bergdolt et al., 2017) further promoting a pro-metastatic environment. In response to C5a in fact, C5aR1 interacts with TLR2 in osteoblasts, promoting the upregulation of CXCL10 (Kwak et al., 2008; Mödinger et al., 2018), a chemokine that is critical for bone cancer cells recruitment, to support osteoclast differentiation and to promote the formation of osteolytic bone metastases (Lee et al., 2012).

Beside the direct actions on the bone, C5a/C5aR axis also exerts indirect pro-metastatic effects by inducing C5a-dependent recruitment of PMN-MDSCs (Corrales et al., 2012) that has been observed to facilitate metastasis. PMN-MDSCs can in fact suppress effector CD8^+^ and CD4^+^ T-cells responses in the lungs and livers of mice with breast malignancy (Vadrevu, 2014) and undergo NETosis. C5a enhances PMN-MDSC migration and invasion and, together with the costimulatory factor nuclear protein high mobility group box 1 (HMGB1) produced by cancer cells, induces the formation of NETs that in turn promote cancer cell dissemination and lung metastasis (Ortiz-Espinosa et al., 2022). Since NETs levels were shown to be elevated in multiple advanced cancer patients (Tohme et al., 2016; Rayes et al., 2019), further studies should be pursued to understand more in depth the contribution of C5a/C5aR1 axis and NETosis specifically during skeletal colonization.

## C5a/C5aR targeting pharmacological approaches

Activation of the complement system is a major pathogenic event that drives various inflammatory responses in numerous diseases. For this reason, a large number of anti-complement drugs are in development, providing tools for blocking specific complement activation pathways, or isolated complement fragments, such as C5a (Floege and Feehally, 2013; Thurman and Le Quintrec, 2016; Thurman and Yapa, 2019).

Among the drugs targeting the C5a/C5aR1/C5aR2 axis that have reached the clinical phases of development for the treatment of various immunological disorders, some are still under evaluation in clinical trials (e.g., Zimura, Nomacopan, Tesidolumab and MOR-210), while some of them have been discontinued (e.g., PMX-53, MEDI-7814, Olendalizumab and others). Approved for clinical use is avacopan, a selective C5a receptor inhibitor, that has been tested for the treatment of ANCA-associated vasculitis with positive results (Jayne et al., 2017). Another available approach to block C5a biological activity is eculizumab, a monoclonal antibody targeting C5 that thus prevents the generation of both C5a and the terminal complement complex (Volokhina et al., 2015). Notably, a trial with eculizumab has been conducted in RA patients (Sadik et al., 2018), and the results from phase II suggested that inhibiting C5 might be a promising approach for the treatment of this disease. These data are in contrast with those of another study reporting that C5aR blockade by PMX-53 in RA patients failed to reduce effectively synovial inflammation (Vergunst et al., 2007), implying that further investigations are necessary to fully explore the role of C5a-C5aR inhibition in human RA.

Preclinical data have also shown that antagonizing C5aR1 after bone fracture in rats by a single application of PMX-53 immediately reversed the negative effect of the trauma-induced systemic inflammation on fracture healing outcome. However, when inhibiting C5aR1 in the early inflammatory phase in a model of uneventful fracture healing with no additional traumatic injury, bone regeneration was unaffected (Takayanagi, 2012). Due to the paucity of data and in light of the fact that PMX-53 has being discontinued, further pre-clinical and clinical studies with novel drugs targeting C5a/C5aR axis for the treatment of fracture healing would be very useful.

Given the involvement of C5a/C5aR1 axis in the development of bone metastasis, studies have also aimed at finding the effect of C5aR1 inactivation in this context. In a syngeneic model of breast cancer for example, C5aR knockout mice or pharmacologic inhibition of C5aR1 reduced lung and liver metastatic burden, while CD8 T cells and inhibiting regulatory T cells were increased. In contrast, there was no significant effect on the growth of primary breast tumors (Ajona et al., 2018a). Moreover, both genetic ablation and pharmacological inhibition of C5a decreased bone metastasis in an *in vivo* mouse cancer model (Ajona et al., 2018b). Interestingly, incubation with DF3016A, a C5aR inhibitor, has been shown to diminish osteoclast-resorbing activity *in vitro* (D'Angelo et al., 2020). Thus, it has been suggested that DF3016A may be used as a potential double-edged blade treatment to fight bone metastases from several tumors, as it can both decrease the osteoclast activity required for the formation of the bone metastatic niche and act at the level of tumor cells by reducing their homing to bone (D'Angelo et al., 2020). Finally, blocking C5aR signaling promotes the anti-tumor efficacy of PD-1/PD-L1 blockade, while the combined immunotherapy based on C5a and PD-1 blockade has shown synergistic effects on both lung cancer growth and metastatic progression (Ajona et al., 2017).

Notably, from the available clinical data, chronic therapies with antagonists of the C5a/C5aR1/C5aR2 axis did not show consistent evident adverse effects on bone density and bone formation when administered in adult patients (Eschbach, 2000; Takata et al., 2004; ClinicalTrials.gov, 2021), thus suggesting that the use of C5 antagonists — even chronically — for related pathologies during post-natal/adult life can be relatively safe from the bone/skeletal point of view.

## Conclusion

Growing evidence has demonstrated the role that C5a-C5aR1 axis plays in mediating the interactions between skeletal and immune systems, both in physiological conditions and in the pathogenesis of several bone inflammatory disorders. Thus, the combined used of standard therapies and of inhibitors of C5a-C5aR1 axis might be a successful strategy for the treatment of bone pathologies in which inflammation and complement system are known to be crucially involved, as rheumatoid arthritis in particular, for which also clinical trials have been conducted using C5aR1 inhibitors, but also for osteopenia and osteoporosis, fracture healing and metastatic bone disease. First preclinical and clinical data indicate that this approach has promises for all these conditions. Taking advantage of the numerous C5aR1 inhibitory compounds that are already available — and even approved for the clinical application — further studies are urgently needed to deeply investigate the effects of such approaches in the treatment of bone inflammatory conditions.
